# Obtaining Bixin- and Tocotrienol-Rich Extracts from Peruvian Annatto Seeds Using Supercritical CO_2_ Extraction: Experimental and Economic Evaluation

**DOI:** 10.3390/foods13101549

**Published:** 2024-05-16

**Authors:** Fiorella P. Cárdenas-Toro, Jennifer H. Meza-Coaquira, Gabriela K. Nakama-Hokamura, Giovani Leone Zabot

**Affiliations:** 1 Section of Industrial Engineering, Department of Engineering, Pontifical Catholic University of Perú, Av. Universitaria 1801, Lima 15088, Peru; jhmeza@pucp.edu.pe (J.H.M.-C.); nakama.gk@pucp.edu.pe (G.K.N.-H.); 2 Laboratory of Agroindustrial Processes Engineering (LAPE), Federal University of Santa Maria (UFSM), 3013, Taufik Germano Rd, Cachoeira do Sul 96503-205, RS, Brazil; giovani.zabot@ufsm.br

**Keywords:** annatto, bixin, tocopherols, tocotrienols, supercritical fluid extraction, cost of manufacturing

## Abstract

Currently, *Bixa orellana* L. extracts are used as a color source in the food, pharmaceutical, and cosmetic industries because they are important as a potential source of antioxidant activity. The extraction is carried out by conventional methods, using alkaline solutions or organic solvents. These extraction methods do not take advantage of the lipid fraction of annatto (*Bixa orellana* L.) seeds, and the process is not friendly to the environment. In this work, the objective was to obtain an extract rich in nutraceuticals (bixin and tocols) of high antioxidant power from Peruvian annatto seeds as a potential source for a functional food or additive in the industry using supercritical fluid extraction (SFE). Experiments related to extraction yield, bixin, tocotrienols, tocopherols, and antioxidant activity were carried out. The SFE was performed at 40 °C, 50 °C, and 60 °C, and 100, 150, and 250 bar with 0.256 kg/h carbon dioxide as the supercritical solvent (solvent-to-feed ratio of 10.2). Supercritical extraction at 60 °C and 250 bar presented the best results in terms of global extraction yield of 1.40 ± 0.01 g/100 g d.b., extract concentration of 0.564 ± 0.005 g bixin/g extract, 307.8 mg α-tocotrienol/g extract, 39.2 mg β-tocotrienol/g extract, 2 mg γ-tocopherol/g extract, and IC_50_ of 989.96 μg extract/mL. Economical evaluation showed that 60 °C, 250 bar, and 45 min presented the lowest cost of manufacturing (2 × 2000 L, COM of USD 212.39/kg extract). This extract is a potential source for functional food production.

## 1. Introduction

Annatto (*Bixa orellana* L.) has been cultivated since pre-Hispanic times in Central America and the Amazon. It has been used as a colorant, medicine, and food ingredient. Annatto belongs to the Bixaceae family, and it is native to tropical America, possibly the southwestern Amazon. This plant is shrubby and reaches heights up to 5 m, and its leaves are black-green and oval or heart-shaped. The fruit is an ovoid capsule that can be red, orange, green, yellow, or a combination of these colors with a reddish color in the outer coat of the annatto seed [1,2].

Annatto seeds contain two pigments with industrial applications, bixin and norbixin, the first being a high-cost dye used in the food and cosmetic industry due to the growing popularity of the natural colorants in the market. For example, the growth of the food coloring market is expected to be 3.7% annually from 2023 to 2025. This is mainly due to the increased awareness of environmental and health issues on the part of consumers and companies. For this reason, the global market for food antioxidants is also growing at an annual rate of 6% from 2019 to 2024. Asia is the region with the greatest growth in this market, and Europe is the fastest-growing region. In addition, there are new regulations that seek to reduce the production of chemical antioxidants. Among the patents registered as *Bixa orellana* during the last 5 years, there have been 61 inventions, of which 87% correspond to health, cosmetics, and food topics and include patents related to annatto oil. This panorama allows for seeing the importance of this research as a scientific and practical contribution in this context [3,4].

**Figure 1:**
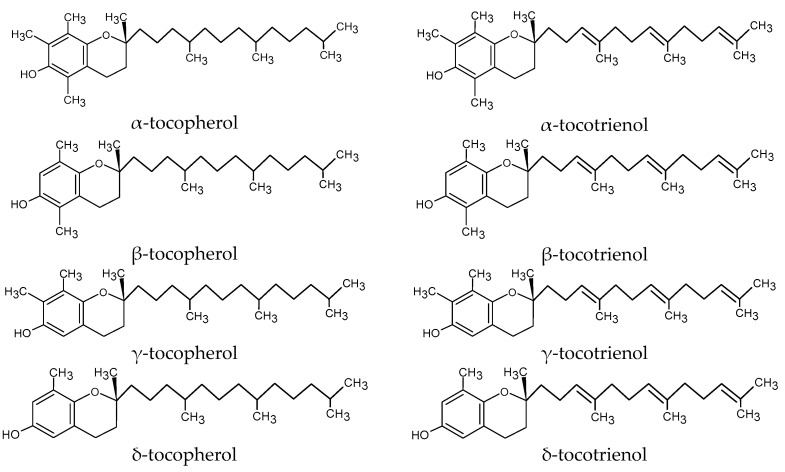
Chemical structure of α-, β-, γ-, and δ-tocopherols and tocotrienols (based on [6]).

One of the byproducts of the industrial production of bixin is an oily fraction enriched with vitamin E [1,2]. Vitamin E includes α-, β-, γ-, and δ-tocopherols and α-, β-, γ-, and δ-tocotrienol, and it is found in the oily fraction of nuts and oil seeds. Vitamin E isoforms have similar chemical structures in terms of a chromanol ring with a hydroxyl group, while α-tocopherol is reported to be the most biologically active, as it is retained at high levels in plasma and tissue compared with other isoforms. β-Tocopherol and α-tocotrienol present 50% and 30% α-tocopherol equivalents, respectively [5]. Tocopherols present a saturated side chain, while tocotrienols present three double bonds in the side chain, conferring a high antioxidant activity [1]. Figure 1 shows the chemical structure of α-, β-, γ-, and δ-tocopherols and tocotrienols [6].

Studies have been carried out to evaluate the extraction performance of bixin and tocols (tocopherols and tocopherols) by conventional methods and by supercritical fluids from Brazilian annatto seeds, obtaining a high content of bixin by conventional methods; however, by supercritical technology, a high percentage of tocotrienols are obtained compared to traditional methods, especially δ-tocotrienol, which is a powerful antioxidant [7,8].

Albuquerque and Meireles [7] studied the defatting of Brazilian annatto seeds with supercritical CO_2_ extraction at selected process conditions of temperatures of 40 and 50 °C, pressure of 20, 31, and 40 MPa, and S/F ratio of 35. The maximum recoveries of δ- and γ-tocotrienol in the extract were 14.6 g/100 g extract and 1.97 g/100 g extract, respectively, at 40 °C and 20 Mpa. The bixin content varied between 1.6 and 4 g bixin/100 g seed d.b.

Zabot et al. [9] proposed a process integration for tocotrienol-rich extracts using supercritical fluid extraction and bixin-rich extracts from Brazilian annatto seeds using low-pressure solvent extraction at a pilot scale to provide an alternative for industrial applications. In this study, the supercritical CO_2_ extraction of annatto seeds at 40 °C, 20 Mpa, and an S/F ratio of 8.9 resulted in a global yield of 2.9 ± 0.01 g extract/100 g seed d.b., with recoveries of 14.85 g/100 g extract and 2.06 g/100 g extract of δ- and γ-tocotrienol, respectively. Additionally, it was found that an extraction time larger than an S/F of 8.9 results in a high consumption of solvent with a low increase in extraction yield.

Vardanega et al. [10] proposed an extraction process of geranylgeraniol and tocotrienols from Brazilian annatto seeds using two sequential steps for the recovery of a geranylgeraniol-rich fraction at 60 °C and 10 Mpa (S/F = 30) followed by a recovery of a tocotrienol-rich fraction at 40 °C and 20 Mpa (S/F = 18), considering the effect of CO_2_ density on the selective extraction of bioactive compounds. In the first step, an extract was obtained with a concentration of 408 ± 21 mg geranylgeraniol/g of extract and 44 ± 3 mg tocotrienol/g of extract. In the second step, an extract was obtained with a concentration of 132 ± 17 mg geranylgeraniol/g of extract and 226 ± 12 mg tocotrienol/g of extract.

Oliveira et al. [1] showed that extracts from Brazilian annatto seeds have antioxidant activity and can be used in the pharmaceutical, cosmetic, and food industries. Additionally, there were no studies related to research on tocotrienol-rich extracts obtained from Peruvian annatto seeds using supercritical CO_2_ extraction. Nolasco-Chumpitaz et al. [11] studied the morphological characterization and bixin content of Peruvian annatto seeds from seven departments in Perú and reported bixin contents between 0.895 and 3.997 g/100 g. Another report from the Peruvian National Institute of Agricultural Innovation showed that bixin content in annatto seeds from the national germplasm bank varied between 3.55% and 4.55%. The content of tocotrienols or tocopherols was not quantified in these studies. The present work, unlike previously published works, addresses the use of Peruvian annatto seeds in which a simultaneous technical and economic evaluation was developed. Thus, the objective of this work was to investigate the influence of supercritical CO_2_ extraction on the global extraction yield, the chemical composition of bixin, tocotrienols, and tocopherols, and the antioxidant activity of extracts from Peruvian annatto seeds, and its economical evaluation under various scenarios.

## 2. Materials and Methods

### 2.1. Materials and Reagents

Annatto seeds were purchased from AgroBeans Company (Lima, Perú). The seeds were placed in plastic bags under vacuum and stored in the freezer at −8 °C protected from light. Carbon dioxide with 99.99% purity was purchased from Praxair Perú S.R.L. (Lima, Perú). Gallic acid monohydrate standard (≥98%) was purchased from Sigma-Aldrich (Saint Louis, MO, USA). A standard mixed solution of α-, β-, γ-, and δ-tocopherols and tocotrienols was purchased from Chromadex Company (Los Angeles, CA, USA). All the solvents and reagents used in the analysis were of analytical grade.

### 2.2. Characterization of Annatto Seeds

Annatto seeds were characterized in terms of proximate composition: moisture, ash, and protein [12]. The lipid content was determined by Soxhlet extraction of 40 g d.b. annatto seeds with 400 mL of hexane for 8 h for further comparison with supercritical fluid extraction. All analyses were performed in duplicate. The annatto seeds were not milled because it was considered that oil and bixin are mainly found in the seed coat, as a previous study reported that there was no difference in bixin yield using milled or whole seeds [13]. The results are given as the mean ± range, where range is the difference between maximum and minimum value.

### 2.3. Extraction Experiments

#### Supercritical Fluid Extraction (SFE)

**Figure 2:**
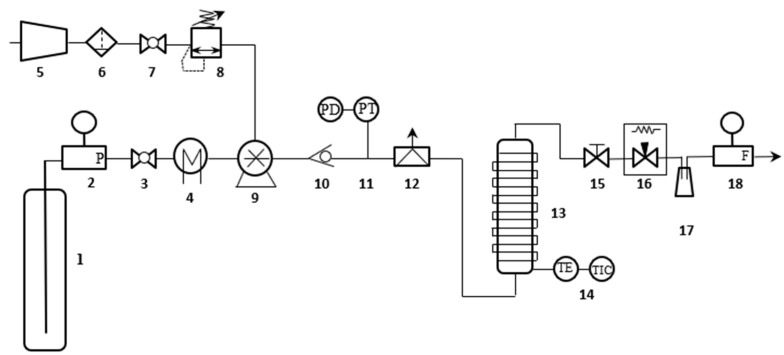
Scheme of supercritical fluid extraction equipment SFT-150 (Supercritical Fluid Technologies, Inc., Newark, NJ, USA). (1) CO_2_ cylinder, (2) manometer, (3) ball valve, (4) CO_2_ pre-chiller, (5) air compressor, (6) air filter, (7) ball valve, (8) relieving regulator, (9) liquid CO_2_ pump, (10) check valve, (11) pressure transmitter PT/pressure differential PD, (12) inline safety head & rupture disc assembly, (13) vessel with preheater assembly, (14) sensor temperature TE/temperature indicator controller TIC, (15) block valve, (16) micrometer valve, (17) glass flask, (18) gas flowmeter.

The extraction experiments were performed in a commercial SFE unit (SFT150, Supercritical Fluid Technologies, Inc., Newark, NJ, USA) equipped mainly with a CO_2_ cylinder, pre-chiller, liquid CO_2_ pump, 100 mL extraction vessel with preheater assembly, flask, and gas flowmeter. A schematic diagram of the equipment is shown in Figure 2. Approximately 50 g of annatto seeds were placed inside a 100 mL extraction vessel and placed inside the preheater assembly. The assembly was heated close to the selected temperature, and then, the CO_2_ was cooled by the refrigerant-based chiller and pumped into the extraction vessel until the selected pressure and temperature (~10 min). Then, the system was kept static for 15 min before the dynamic extraction step. For dynamic extraction, a micrometric valve was opened at a constant CO_2_ flow rate of 0.256 kg/h (5 ft^3^/h) for 2 h. The extract was collected inside a 40 cm^3^ amber glass flask immersed in an ice bath. The extracted mass was measured in an analytical balance (LX 220 A, Precisa Gravimetrics AG, Dietikon, Switzerland) at time intervals of 5 min, 10 min, 20 min, 30 min, 45 min, 60 min, 90 min, and 120 min and collected in the flask. The final extract was stored under freezing (−4 °C) in the absence of light for further analyses.

The experiments were carried out at temperatures of 40, 50, and 60 °C and pressures of 100, 150, and 250 bar in duplicate. Pressures higher than 250 bar were not used in the present study because they exceeded the maximum capacity of the extraction system. Table 1 shows that CO_2_ density varied between 290 and 879.5 kg/m^3^ [14].

### 2.4. Extract Characterization

#### 2.4.1. Global Extraction Yield

The global extraction yield (X_0_) was calculated as the amount of the extract (m_extract_) that can be recovered from raw material on a dry basis (m_sample_) at a given pressure and temperature, according to Equation (1). The results were expressed as g extract/100 g d.b. The results are given as the mean ± range.

X_0_ (g extract/100 g d.b.) = m_extract_ (g) × 100/m_sample_ (g) d.b. (1)

#### 2.4.2. Analysis of Bixin

The determination of the bixin content in extracts was performed according to the Joint FAO/WHO Expert Committee on Food Additives Specifications (2006) [15]. The extracts (approximately 2 mg of each sample) obtained by Soxhlet and supercritical fluid extraction were diluted in acetone, vortexed, and placed in quartz cells for reading in a UV-VIS spectrophotometer (G10S UV-VIS, Thermo Fisher Scientific, Pudong, Shanghai, China). The absorbance was measured at 487 nm. The bixin content was determined using the Lambert-Beer law (Equation (2)), where A= absorbance of sample, V_i_ = dilution volume (mL), V_i_’ = volume of aliquot for dilution (mL), E_1 cm_^1%^ = specific absorbance = 3090, m = extract mass (µg). The results were expressed as g bixin/g extract. The results are given as the mean ± range.

Bixin (g bixin/g extract) = (A × 10^4^ × Vi)/(E_1 cm_^1%^ × m × V_i_’) (2)

#### 2.4.3. Analysis of Tocopherols and Tocotrienols

Each sample was extracted twice using 2 mL of n-hexane. The combined extracts were taken to dryness under nitrogen, and the residue was reconstituted in a volume of 1.5 mL with n-hexane. The extract was dried with 0.5 g of anhydrous sodium sulfate, centrifuged at 4000× *g* for 20 min at 1 °C, and transferred to a dark vial for subsequent HPLC analysis. Samples were separated using a normal phase HPLC column on a Waters 2695 Separation Module (Waters, Milford, MA, USA) equipped with a Waters 2475 multifluorescence detector and Empower software 2. A YMC-Pack Silica Column (3 μm, 250 × 4.6 mm column (Kyoto, Japan)) and a 4.0 × 2.0 mm guard column were used for tocopherol separation at 35 °C. The mobile phase was composed of n-hexane/2-propanol/acetic acid (1000/6/5, *v*/*v*/*v*). A solvent flow rate of 1.4 mL/min under isocratic conditions was used. Seven microliters of the sample were injected. The fluorescence detector was programmed at excitation and emission wavelengths of 290 and 330 nm, respectively. Tocopherols/tocotrienol were identified and quantified by comparing their retention time to known previously injected standards. The results were expressed as mg/g extract.

#### 2.4.4. Antioxidant Activity by DPPH Free-Radical Scavenging Assay

The DPPH free-radical scavenging assay activity of extracts was determined according to previous methods with a slight modification [16,17]. A 20 mg/L DPPH working solution with methanol was prepared daily and stored in the freezer until needed. The standard curve was prepared using Trolox in the range of 12 to 1000 μM. Extract solutions were prepared by diluting 0.0075 g of Soxhlet or supercritical fluid extracts in methanol, followed by successive dilutions to obtain concentrations of 25 to 2500 μg extract/mL. Then, an aliquot of 0.1 mL of sample (extract or standard) was added to 3.9 mL of DPPH solution. The reaction mixture was stirred vigorously and allowed to react for 30 min, and then, the absorbance (A_1_) was measured at 515 nm in a UV-VIS spectrophotometer (G10S UV-VIS, Thermo Fisher Scientific, Pudong, Shanghai, China). The initial absorbance of 0.1 mL methanol (blank) and 3.9 mL of DPPH solution (A_0_) was taken at minute zero. Each sample was measured in duplicate.

The ability to scavenge DPPH radicals was expressed as the inhibition percentage calculated by the following Equation (3):

% inhibition = (1 − A_1_/A_0_) × 100% (3)

where A_0_ is the absorbance of the blank at minute zero, and A_1_ is the absorbance of the sample at 30 min.

A linear calibration curve was produced with R^2^ = 0.9946 (% inhibition = 0.0875 × Trolox concentration (μM) + 2.3094).

The IC_50_ was calculated from the equation of line obtained by plotting a graph of extract concentration versus % inhibition.

### 2.5. Statistical Analysis

An analysis of variance (*p* < 0.05) was used for the comparison of different global extraction yields and bixin contents in extracts under the studied conditions. The different results were evaluated using Tukey’s test.

### 2.6. Economic Evaluation of SFE Process

**Figure 3:**
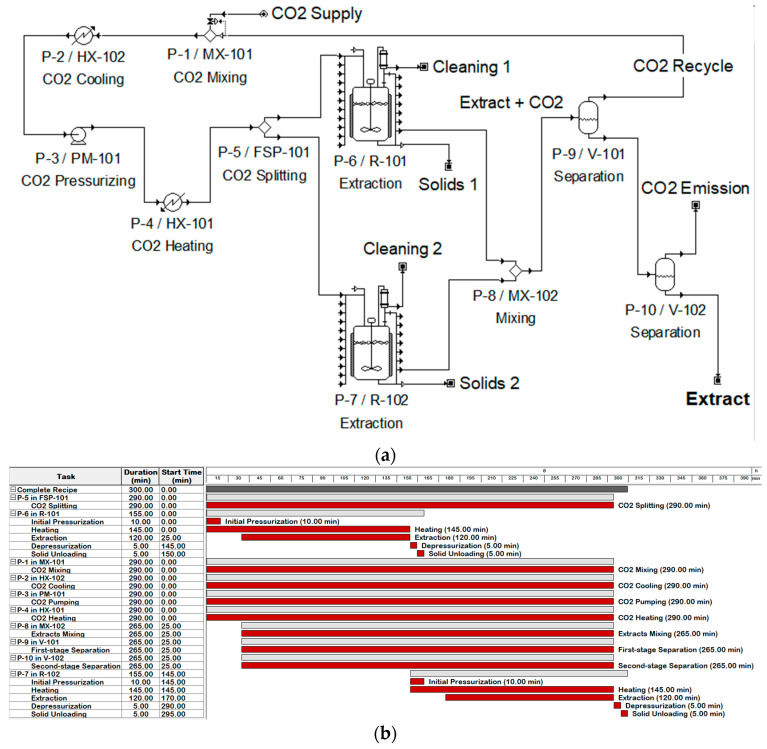
(**a**) Process flowsheet and (**b**) a Gantt chart for an extraction time of 120 min.

The economic evaluation was performed in the SuperPro Designer 9.0^®^ software (Intelligen Inc., Scotch Plains, NJ, USA) using the experimental data of the SFE process. The procedures include mainly annatto loading, bed pressurization with CO_2_, bed heating, oil extraction, bed depressurizing, and bed unloading. For representation purposes, a flowsheet and a Gantt chart for an extraction time of 120 min are presented in Figure 3. The SFE process was designed to operate for 7920 h per year, which corresponds to 3 daily shifts (8 h each shift) for 330 days per year. The yearly remaining time was considered for cleaning and equipment maintenance.

Commonly, equipment purchase costs for one or two discrete sizes are available. Alternatively, costs for other equipment sizes or capacities must be estimated. Scaling the equipment cost to the required capacity is possible via the power law (Equation (4)), where C_1_ is the equipment cost with capacity Q_1_, C_2_ is the known base cost for equipment with capacity Q_2_, and M is a constant depending on the equipment type [18,19,20]. Values of M were obtained from the literature because the cost of a specific item is a function of size, materials of construction, design pressure, and design temperature [18,19,20,21,22]. The base costs were acquired in 2023 (local quotation). Two capacities of an SFE plant with two extractor vessels were evaluated: 200 L (USD 1.356 mi) and 2000 L (USD 5.401 mi). The plants include buildings, engineering, construction, installation, and all accessories/equipment to operate. Other input data, such as the cost of raw materials, wages, and utilities, are presented in Table 2.

C_1_ = C_2_ × (Q_1_/Q_2_)^M^ (4)

The cost of manufacturing (COM) of bioactive extracts depends on the sum of three main components: direct manufacturing costs (i.e., seeds, water, and CO_2_), fixed manufacturing costs (i.e., equipment), and general expenses (i.e., management costs and research & development). Capital costs associated with buildings and equipment and other costs, such as storage of raw materials and extracts, electrical facilities, instrumentation, engineering, and management fees, were considered. The waste generated after performing the SFE process can be considered harmless and clean. The COM was simulated for each point of annatto seed extracts obtained in the experimental assay (a total of 8 points for each condition). The objective of this simulation along the time was to have a response of the economically feasible time that is suitable for performing the extraction based on extract yield.

The yields and composition were assumed to have the same performance on larger scales as the findings obtained on the laboratory scale. The percent contribution of the itemized costs (fixed capital investment—FCI, cost of raw materials—CRM, cost of operational labor—COL, and cost of utilities—CUT) was evaluated considering the most feasible time.

## 3. Results and Discussion

### 3.1. Characterization of Annatto Seeds

Table 2 shows the results for annatto seed characterization. The moisture, protein, and ash were 11.26 ± 0.07%, 15.62 ± 0.11%, and 4.93 ± 0.04%, respectively. The lipid content was 3.04 ± 0.01%. The content of lipids was lower than those obtained by other studies for Brazilian annatto seeds, as shown in Table 3.

### 3.2. Global Extraction Yield

Table 4 shows the global extraction yield in extracts obtained under the supercritical studied conditions. Global extraction yields varied between 0.14 ± 0.00 and 1.42 ± 0.04 g/100 g seed d.b. at the studied pressures (100, 150, and 250 bar) and temperatures (40, 50, and 60 °C) at a fixed CO_2_ rate of 0.256 kg/h for 2 h (S/F of 10.2). High extraction yields were obtained at operating conditions of 150 bar and 40 °C (1.42 ± 0.04 g/100 g d.b.), 150 bar and 50 °C (1.35 ± 0.03 g/100 g d.b.), and 250 bar and 60 °C (1.40 ± 0.01 g/100 g d.b.), while the lowest extraction yield was obtained at 100 bar and 60 °C (0.14 ± 0.00 g/100 g d.b.). The extraction yields obtained with hexane and ethanol were 3.15 ± 0.1 g/100 g d.b. and 6.45 ± 0.10 g/100 g d.b., respectively. These values were higher than those obtained with supercritical extraction experiments, mainly due to the longer extraction times (6 h) when compared to supercritical extraction. Albuquerque and Meireles [7] reported an increase in global yield with an increase in temperature from 40 °C (1.8 ± 0.2 g/100 g seed d.b.) to 50 °C (2.07 ± 0.1 g/100 g d.b.) at 200 bar and an S/F of 35. Vardanega et al. [10] reported an increase in global yield increasing temperature from 40 °C (2.3 ± 0.1 g/100 g seed d.b.) to 60 °C (2.4 ± 0.1 g/100 g d.b.) at 170 bar and an S/F of 25. These two works did not report results for 100 bar.

**Figure 4:**
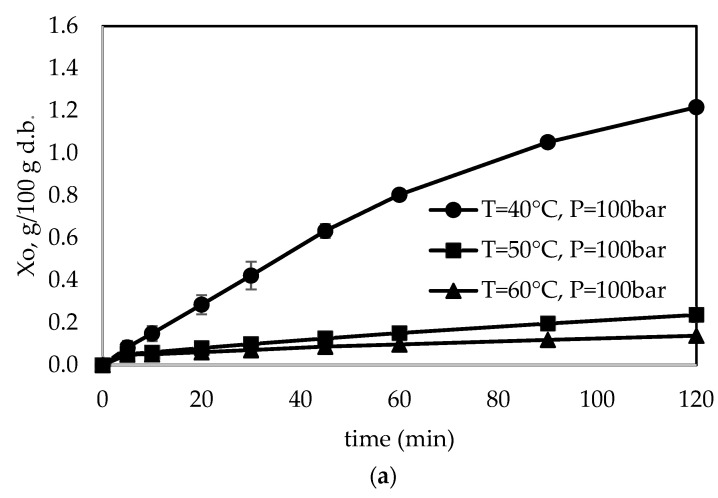

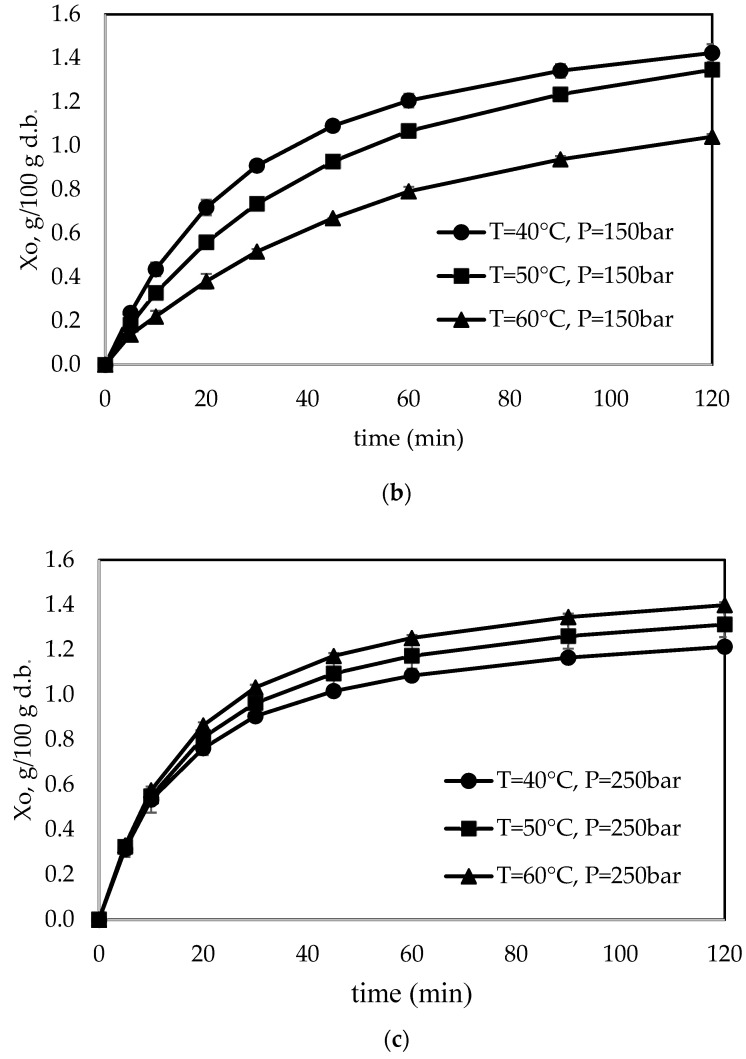
Overall extraction curve (OEC) for the studied conditions: (**a**) 100 bar, (**b**) 150 bar, and (**c**) 250 bar.

Figure 4 shows the overall extraction curve (OEC) for the studied conditions. It presents three different regions: a constant extraction rate (CER) period where the mass transfer is controlled by the convection mechanism; a falling extraction rate (FER) period where convection and diffusion mechanisms are important; and a diffusion-controlled (DC) period where the mass transfer is controlled by the diffusion mechanism [23]. There was an increase in the global extraction yield when the pressure increased from 100 to 250 bar at a temperature of 60 °C. In addition, an increase in global extraction yield was observed with an increase in pressure from 100 to 150 bar at temperatures of 40 and 50 °C, as well as a slight decrease in extraction yield when the pressure increased from 150 to 250 bar. Additionally, we observe that if we increase the pressure from 100 to 250 bar, the diffusion-controlled period is reached in a shorter time.

### 3.3. Bixin Content in Extracts

Table 5 shows the bixin content in extracts obtained under the supercritical studied conditions. The bixin content in the supercritical extracts varied between 0.031 ± 0.000 and 0.577 ± 0.026 g bixin/g extract for the studied conditions. High bixin content in extracts was obtained at 50 °C and 250 bar, and 60 °C and 250 bar, while low bixin content in extracts was obtained at 40 °C and 100 bar, 50 °C and 100 bar, 60 °C and 100 bar, and 60 °C and 150 bar. It was observed that at a constant temperature of 40 °C, 50 °C, and 60 °C, an increment in pressure resulted in an increment of the bixin content in supercritical extracts. At a constant pressure of 100 bar and 150 bar, an increment in temperature from 40 °C to 60 °C resulted in a decrement in the bixin content. On the other hand, at 250 bar, an increase in temperature produced an increase in the bixin content. The bixin contents of Soxhlet extracts obtained with hexane (1.145 ± 0.100 g bixin/100 g extract) and ethanol (18.754 ± 0.247 g bixin/100 g extract) were higher than those obtained in the supercritical extracts. This is mainly due to the extraction time (6 h) and the increase in the polarity of the solvent ethanol. Pingale [24] reported a higher percentage of bixin in ethanol extracts (8.09%).

### 3.4. Tocotrienol and Tocopherol Contents in Extracts

Table 6 shows the tocotrienol and tocopherol contents in extracts obtained at 150 and 250 bar. These conditions showed higher global extraction yield and higher bixin content in extracts than yields obtained at 100 bar. For all supercritical extracts, the content of δ-tocotrienol was higher than that of γ-tocotrienol and β-tocopherol. This tendency was like those reported in other work [25]. High values of δ-tocotrienol, γ-tocotrienol, and β-tocopherol were obtained at 250 bar and 40 °C and 250 bar and 60 °C. Soxhlet extractions resulted in 210.7 mg δ-tocotrienol/g extract, 20.6 mg γ-tocotrienol/g extract, and 2.2 mg β-tocopherol/g extract for hexane; and 169.0 mg δ-tocotrienol/g extract, 19.5 mg γ-tocotrienol/g extract and 2.18 mg β-tocopherol/g extract for ethanol. The values of γ- tocotrienol and β-tocopherol were similar for Soxhlet and supercritical extracts; however, supercritical extracts showed higher concentrations of δ-tocotrienol than Soxhlet extracts. This behavior was also reported for supercritical extracts from Brazilian annatto seeds [7]. Additionally, the presence of β-tocopherol was not detected in the extracts obtained by supercritical extraction at 150 bar.

### 3.5. Antioxidant Activity by DPPH Free-Radical Scavenging Assay in Extracts

**Figure 5:**
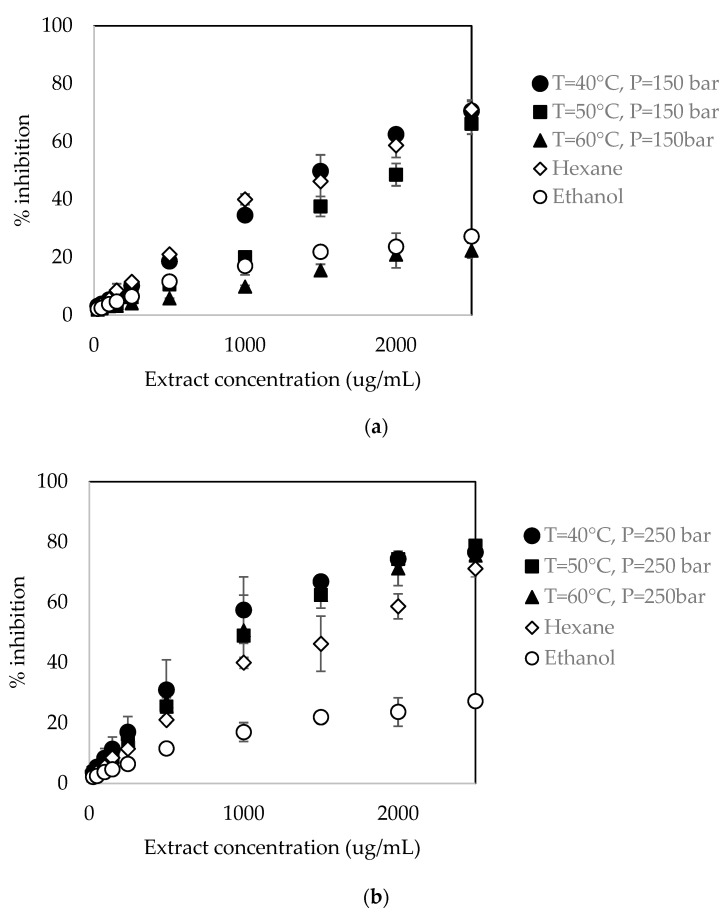
Percentage of inhibition in supercritical extracts at (**a**) 150 bar and (**b**) 250 bar and its comparison with Soxhlet extractions.

Figure 5 shows the percentages of inhibition obtained for each extract concentration at pressures of 150 and 250 bar, which confirms that supercritical extracts capture the DPPH free radicals present in the methanolic solution. A higher concentration of extract resulted in a greater antioxidant capacity, which suggests that the percentage of inhibition of DPPH radical had a direct relationship with the extract concentrations in the range of 25 to 2500 μg/mL for 150 bar and 250 bar. Similar behavior was found for extracts obtained by Soxhlet extraction with hexane and ethanol with percentages of inhibition up to 71 and 21, respectively.

Table 7 shows the IC_50_ values obtained in the supercritical extracts. IC_50_ is the amount of extract necessary to reduce the concentration of DPPH free radicals by 50%. The lowest value to reduce the concentration of DPPH free radicals by half was 818.51 μg extract/mL, which corresponded to 40 °C and 250 bar. The IC_50_ values for the extracts obtained at 250 bar were lower than those obtained at 150 bar, indicating the high antioxidant capacity of extracts obtained at 250 bar. This finding is because the concentrations of bixin, tocopherol, and tocotrienol recovered in extracts increased with the increase in pressure from 150 to 250 bar. The IC_50_ values for the Soxhlet extracts were 1482.94 μg/mL for hexane and 4451.1 μg/mL for ethanol. The extract obtained by hexane (1644.74 μg/mL) had similar IC_50_ values to those obtained by the supercritical extraction, possibly due to the higher concentration of tocotrienols obtained with this solvent compared to ethanol (4450.98 μg/mL).

### 3.6. Cost of Manufacturing of Extracts

**Figure 6:**
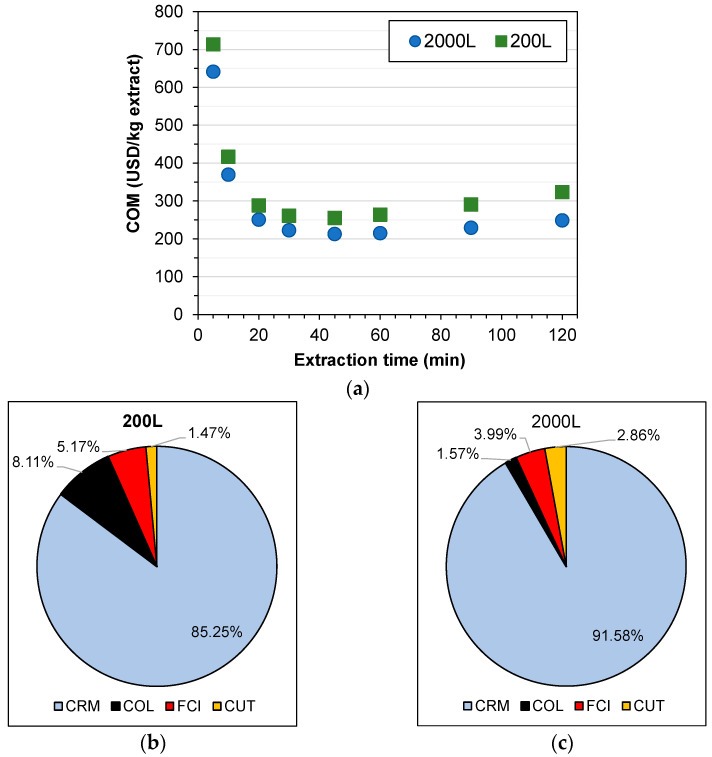
(**a**) Kinetic behavior of COM for 200 L and 2000 L plants for the scenario of temperature of 60 °C and pressure of 250 bar; pie charts for (**b**) 200 L and (**c**) 2000 L plants for the scenario of temperature of 60 °C, pressure of 250 bar, and extraction time of 45 min.

The results of COM and itemized costs for both 200 L and 2000 L plants are presented in Table 8 and Table 9 and in Figure 6. The smallest COM (USD 212.39/kg extract) was obtained in the condition of 250 bar, 60 °C, and 45 min for the 2000 L plant. In this scenario, it is possible to reach a productivity of 79,586 kg of extracts per year. Based on Figure 6, according to the economic results, the best time for extraction is nearly 45 min, thus not needing complete recovery of extracts on larger times (>120 min). In all scenarios, the defatted seeds were considered for being used for further bixin extraction. The main contribution to the COM was the CRM, which was around 70–90%. In such cases, the main aspect for reducing the COM is negotiating a lower acquisition cost of raw materials, especially the annatto seeds and the industrial CO_2_. The high impact of raw materials on the COM of many extracts is also reported elsewhere [26,27,28]. Therefore, the impact of high-pressure equipment on the COM is not the highest, allowing the application of supercritical fluid processes at an industrial scale. These results also demonstrate that the conditions of 60 °C and 250 bar are suitable for obtaining bixin and tocopherol-rich extracts from annatto seeds.

## 4. Conclusions

In this research, a technical and economic evaluation of the supercritical fluid extraction of Peruvian annatto seeds was studied. Supercritical fluid extracts at the highest pressure condition of 250 bar have the highest antioxidant power and highest contents of δ-tocotrienol, γ-tocotrienol, and β-tocopherol compared to extracts obtained by conventional methods. Supercritical extraction is selective since extracts were obtained with different contents of bixin, tocols, and antioxidant capacities at certain pressures and temperatures. According to the results, the supercritical extract obtained at 60 °C and 250 bar presented the best results in terms of global extraction yield of 1.40 ± 0.01 g/100 g d.b., extract concentration of 0.564 ± 0.005 g bixin/g extract, 307.8 mg δ-tocotrienol/g extract, 39.2 mg γ-tocotrienol/g extract and 2 mg β-tocopherol/g extract, and IC_50_ of 989.96 μg extract/mL. Additionally, it is shown that at 60 °C, 250 bar, and 45 min, it is possible to obtain extracts with the lowest cost of manufacturing (2 × 2000 L, COM of USD 212.39/kg extract). It is possible via supercritical technology to obtain extracts rich in nutraceuticals (tocols and bixin) to use as functional foods. With this method, these health-beneficial components can be recovered in a stage before the conventional processing to obtain bixin.

## Figures and Tables

**Table 1:** Density of CO_2_ at studied conditions [14].

T (°C)	P (Bar)	Density (kg/m^3^)
40	100	628.6
40	150	780.2
40	250	879.5
50	100	384.3
50	150	699.8
50	250	834.2
60	100	290.0
60	150	604.1
60	250	786.6

**Table 2:** Input economic parameters used for simulating the COM of annatto seed extracts by SFE.

Economic Parameter	Value	Dimension
Fixed capital investment (FCI)		
Total cost of SFE (200 L) ^a,b^	1,356,000.00	(USD)
Total cost of SFE (2000 L) ^a,b^	5,401,000.00	(USD)
Annual depreciation rate ^c^	10	(%)
Annual maintenance rate ^c^	6	(%)
Project lifetime	25	(years)
Annual time worked	7920	(h/year)
Cost of raw material (CRM)		
Annatto seeds ^d^	3.67	(USD/kg)
Transport and pre-processing of annatto seeds ^e^	40	(USD/ton)
Industrial CO_2_^d^	3.89	(USD/kg)
Water (for direct use in the process) ^d^	4.00	(USD/ton)
Cost of operational labor (COL)		
Wage (with benefits and administration) ^f^	16.80	(USD/h.worker)
Number of workers per shift	2	(Worker/shift)
Total wage per day (3 shifts/day)	806.40	(USD/day)
Cost of utilities (CUT)		
Water (for cooling and cleaning) ^d^	1.00	(USD/ton)
Steam ^d^	12.00	(USD/ton)
Glycol solution ^d^	10.00	(USD/ton)
Electricity ^d^	0.25	(USD/kW.h)

^a^ The design is similar to that one shown in the flowsheet in Figure 3 (2 extraction vessels and 2 separation vessels); ^b^ estimated cost using the power law of capacity (Equation (4)); ^c^ based on [21]; ^d^ direct quotation for the reference year of 2023; ^e^ the pre-processing steps include drying (when needed) and storing the samples until further use; ^f^ [22].

**Table 3:** Characterization of annatto seeds.

Component	Composition (%)		
	This Work	[8]	[7]
Moisture	11.26 ± 0.07	11.5	12.3 ± 0.1
Protein	15.62 ± 0.11	10.34	12.1 ± 0.2
Ash	4.93 ± 0.04	3.82	6.2 ± 0.1
Lipids	3.04 ± 0.01	4.99	3.7 ± 0.0

**Table 4:** Global extraction yield of extracts obtained by supercritical fluid extraction.

T (°C)	P (Bar)	Density (kg/m^3^)	Global Extraction Yield (g Extract/100 g d.b.) *
40	100	628.6	1.22 ± 0.01 ^C^
40	150	780.2	1.42 ± 0.04 ^A^
40	250	879.5	1.21 ± 0.01 ^C^
50	100	384.3	0.24 ±0.03 ^E^
50	150	699.8	1.35 ± 0.03 ^AB^
50	250	834.2	1.31 ± 0.06 ^B^
60	100	290.0	0.14 ± 0.01 ^F^
60	150	604.1	1.04 ± 0.01 ^D^
60	250	786.6	1.40 ± 0.01 ^A^

* The different letters indicate statistically significant differences between the means of pairs compared (Tukey’s test) at a significance level of 0.05.

**Table 5:** Bixin content in supercritical extracts.

T (°C)	P (Bar)	Bixin Content (g Bixin/g Extract) *
40	100	0.036 ± 0.006 ^E^
40	150	0.239 ± 0.008 ^C^
40	250	0.546 ± 0.015 ^B^
50	100	0.031 ± 0.001 ^E^
50	150	0.099 ± 0.006 ^D^
50	250	0.577 ± 0.026 ^A^
60	100	0.031 ± 0.005 ^E^
60	150	0.034 ± 0.001 ^E^
60	250	0.564 ± 0.005 ^AB^

* The different letters indicate a statistically significant difference between the means of pairs compared (Tukey’s test) at a significance level of 0.05.

**Table 6:** Tocotrienol and tocopherol contents in supercritical extracts.

T (°C)	P (Bar)	δ-Tocotrienol (mg/g Extract)	γ-Tocotrienol (mg/g Extract)	β-Tocopherol (mg/g Extract)
40	150	236.9	30.3	n.d.
40	250	312.5	38.7	2.4
50	150	161.0	20.4	n.d.
50	250	232.3	28.3	1.4
60	150	51.4	5.1	n.d.
60	250	307.8	39.2	2.0

n.d.: not detected.

**Table 7:** IC_50_ values obtained in the supercritical extracts.

T (°C)	P (Bar)	IC_50_ (μg/mL)
40	150	1626.53
40	250	818.51
50	150	1997.07
50	250	1066.14
60	150	5573.93
60	250	989.96

**Table 8:** Cost of manufacturing of extracts and itemized costs for a plant of 200 L.

Scenario	T (°C)	P (Bar)	t (min)	COM (USD/kg)	CRM (%)	COL (%)	FCI (%)	CUT (%)
1	40	100	5	2649.39	92.83	4.14	2.63	0.40
2	40	100	10	1528.28	91.79	4.77	3.04	0.40
3	40	100	20	868.53	89.80	5.85	3.73	0.62
4	40	100	30	630.48	88.00	6.83	4.35	0.82
5	40	100	45	470.05	85.57	8.15	5.19	1.09
6	40	100	60	407.75	83.43	9.31	5.93	1.33
7	40	100	90	399.89	80.40	10.66	6.79	2.15
8	40	100	120	367.87	76.91	12.85	8.19	2.05
9	50	100	5	4290.15	92.72	4.13	2.63	0.52
10	50	100	10	3867.66	91.68	4.77	3.04	0.51
11	50	100	20	3038.89	89.70	5.85	3.72	0.73
12	50	100	30	2679.63	87.89	6.82	4.35	0.94
13	50	100	45	2370.15	85.46	8.14	5.18	1.22
14	50	100	60	2157.28	83.32	9.30	5.92	1.46
15	50	100	90	2142.77	80.28	10.64	6.78	2.30
16	50	100	120	1895.82	76.79	12.83	8.18	2.20
17	60	100	5	4585.00	92.62	4.13	2.63	0.62
18	60	100	10	4514.21	91.59	4.76	3.03	0.62
19	60	100	20	4089.62	89.60	5.84	3.72	0.85
20	60	100	30	3789.52	87.79	6.81	4.34	1.06
21	60	100	45	3445.60	85.35	8.13	5.18	1.34
22	60	100	60	3402.06	83.21	9.29	5.92	1.58
23	60	100	90	3291.09	79.60	11.24	7.16	2.00
24	60	100	120	3244.33	76.68	12.82	8.16	2.34
25	40	150	5	992.63	92.75	4.03	2.56	0.66
26	40	150	10	545.05	91.73	4.65	2.96	0.66
27	40	150	20	344.29	89.79	5.85	3.72	0.64
28	40	150	30	294.38	87.97	6.83	4.35	0.85
29	40	150	45	272.94	85.54	8.14	5.19	1.13
30	40	150	60	271.77	83.39	9.30	5.93	1.38
31	40	150	90	288.94	79.77	11.26	7.17	1.80
32	40	150	120	315.01	76.84	12.84	8.18	2.14
33	50	150	5	1287.89	92.64	4.02	2.56	0.78
34	50	150	10	727.58	91.63	4.64	2.96	0.77
35	50	150	20	442.76	89.68	5.84	3.72	0.76
36	50	150	30	352.55	87.87	6.82	4.34	0.97
37	50	150	45	321.72	85.43	8.13	5.18	1.26
38	50	150	60	307.64	83.28	9.29	5.92	1.51
39	50	150	90	314.94	79.66	11.24	7.16	1.94
40	50	150	120	333.58	76.73	12.82	8.17	2.28
41	60	150	5	1702.10	92.52	4.02	2.56	0.90
42	60	150	10	1089.16	91.53	4.63	2.95	0.89
43	60	150	20	654.04	89.58	5.83	3.72	0.87
44	60	150	30	518.72	87.76	6.81	4.34	1.09
45	60	150	45	446.91	85.32	8.12	5.17	1.39
46	60	150	60	415.67	83.17	9.28	5.91	1.64
47	60	150	90	415.87	79.55	11.23	7.15	2.07
48	60	150	120	432.59	76.62	12.80	8.16	2.42
49	40	250	5	744.93	92.71	4.02	2.56	0.71
50	40	250	10	447.61	91.70	4.64	2.96	0.70
51	40	250	20	324.38	89.75	5.84	3.72	0.69
52	40	250	30	296.36	87.92	6.82	4.34	0.92
53	40	250	45	293.18	85.47	8.13	5.18	1.22
54	40	250	60	302.33	83.30	9.29	5.92	1.49
55	40	250	90	333.88	79.66	11.24	7.16	1.94
56	40	250	120	370.91	76.72	12.81	8.16	2.31
57	50	250	5	723.38	92.59	4.02	2.56	0.83
58	50	250	10	436.62	91.59	4.64	2.95	0.82
59	50	250	20	306.28	89.65	5.83	3.72	0.80
60	50	250	30	278.67	87.82	6.81	4.34	1.03
61	50	250	45	272.94	85.36	8.12	5.12	1.34
62	50	250	60	280.67	83.20	9.28	5.91	1.62
63	50	250	90	309.36	79.55	11.22	7.15	2.08
64	50	250	120	343.45	76.60	12.80	8.15	2.45
65	60	250	5	713.12	92.49	4.01	2.56	0.94
66	60	250	10	416.50	91.49	4.63	2.95	0.93
67	60	250	20	287.82	89.55	5.83	3.71	0.91
68	60	250	30	260.42	87.71	6.80	4.33	1.16
69	60	250	45	255.30	85.25	8.11	5.17	1.47
70	60	250	60	263.45	83.08	9.27	5.90	1.75
71	60	250	90	290.35	79.43	11.21	7.14	2.22
72	60	250	120	322.84	76.49	12.77	8.15	2.59

**Table 9:** Cost of manufacturing of extracts and itemized costs for a plant of 2000 L.

Scenario	T (°C)	P (Bar)	t (min)	COM (USD/kg)	CRM (%)	COL (%)	FCI (%)	CUT (%)
1	40	100	5	2396.49	96.22	0.84	2.12	0.82
2	40	100	10	1356.14	95.74	0.97	2.47	0.82
3	40	100	20	754.40	94.60	1.17	2.98	1.25
4	40	100	30	537.73	93.58	1.35	3.44	1.63
5	40	100	45	390.31	92.25	1.59	4.03	2.13
6	40	100	60	331.59	91.12	1.79	4.54	2.55
7	40	100	90	290.07	89.29	2.11	5.36	3.24
8	40	100	120	282.30	87.86	2.36	6.00	3.78
9	50	100	5	3869.66	96.00	0.84	2.12	1.04
10	50	100	10	3414.47	95.53	0.97	2.46	1.04
11	50	100	20	2659.94	94.38	1.17	2.97	1.48
12	50	100	30	2288.76	93.36	1.35	3.43	1.86
13	50	100	45	1969.80	92.03	1.58	4.02	2.37
14	50	100	60	1760.21	90.90	1.78	4.53	2.79
15	50	100	90	1556.76	89.06	2.11	5.34	3.49
16	50	100	120	1455.01	87.63	2.36	5.98	4.03
17	60	100	5	4106.44	95.79	0.83	2.11	1.27
18	60	100	10	4008.71	95.32	0.97	2.45	1.26
19	60	100	20	3554.72	94.17	1.17	2.97	1.70
20	60	100	30	3230.53	93.14	1.35	3.42	2.09
21	60	100	45	2863.16	91.81	1.58	4.01	2.60
22	60	100	60	2765.43	90.67	1.78	4.51	3.04
23	60	100	90	2588.67	88.82	2.10	5.33	3.74
24	60	100	120	2492.33	87.39	2.35	5.97	4.29
25	40	150	5	861.42	96.14	0.83	2.10	0.93
26	40	150	10	467.21	95.67	0.96	2.44	0.93
27	40	150	20	299.31	94.56	1.17	2.98	1.29
28	40	150	30	250.76	93.53	1.35	3.43	1.69
29	40	150	45	226.73	92.18	1.59	4.02	2.21
30	40	150	60	221.11	91.03	1.79	4.53	2.65
31	40	150	90	227.45	89.17	2.11	5.35	3.37
32	40	150	120	241.83	87.73	2.36	5.99	3.93
33	50	150	5	1116.89	95.92	0.83	2.10	1.15
34	50	150	10	622.97	95.46	0.96	2.44	1.14
35	50	150	20	385.27	94.34	1.17	2.97	1.52
36	50	150	30	310.61	93.31	1.35	3.42	1.92
37	50	150	45	267.28	91.96	1.58	4.01	2.45
38	50	150	60	250.46	90.80	1.78	4.52	2.90
39	50	150	90	248.05	88.94	2.10	5.34	3.62
40	50	150	120	256.25	87.49	2.35	5.97	4.19
41	60	150	5	1484.69	95.71	0.83	2.09	1.37
42	60	150	10	938.00	95.24	0.96	2.43	1.37
43	60	150	20	568.23	94.13	1.17	2.96	1.74
44	60	150	30	442.66	93.08	1.35	3.42	2.15
45	60	150	45	371.52	91.73	1.58	4.00	2.69
46	60	150	60	338.61	90.57	1.78	4.51	3.14
47	60	150	90	327.64	88.71	2.10	5.32	3.87
48	60	150	120	332.49	87.26	2.35	5.95	4.44
49	40	250	5	670.87	95.86	0.80	2.03	1.31
50	40	250	10	396.38	95.42	0.93	2.35	1.30
51	40	250	20	282.28	94.48	1.17	2.97	1.38
52	40	250	30	252.52	93.42	1.35	3.42	1.81
53	40	250	45	243.78	92.03	1.58	4.01	2.38
54	40	250	60	246.23	90.85	1.78	4.52	2.85
55	40	250	90	263.02	88.94	2.10	5.33	3.63
56	40	250	120	284.95	87.45	2.35	5.96	4.24
57	50	250	5	651.42	95.64	0.80	2.02	1.54
58	50	250	10	386.82	95.19	0.93	2.35	1.53
59	50	250	20	266.29	94.26	1.17	2.96	1.61
60	50	250	30	237.56	93.19	1.35	3.42	2.04
61	50	250	45	226.97	91.81	1.58	4.00	2.61
62	50	250	60	228.61	90.61	1.78	4.51	3.10
63	50	250	90	243.77	88.70	2.10	5.32	3.88
64	50	250	120	264.03	87.22	2.34	5.95	4.49
65	60	250	5	641.48	95.42	0.80	2.02	1.76
66	60	250	10	369.20	94.98	0.92	2.34	1.76
67	60	250	20	250.39	94.04	1.17	2.96	1.83
68	60	250	30	222.13	92.98	1.34	3.41	2.27
69	60	250	45	212.39	91.58	1.57	3.99	2.86
70	60	250	60	214.65	90.39	1.77	4.49	3.35
71	60	250	90	228.94	88.47	2.09	5.30	4.14
72	60	250	120	248.35	86.98	2.34	5.93	4.75

## Data Availability

The original contributions presented in the study are included in the article, further inquiries can be directed to the corresponding author.
